# Environmental Cycles, Melatonin, and Circadian Control of Stress Response in Fish

**DOI:** 10.3389/fendo.2019.00279

**Published:** 2019-06-11

**Authors:** Francisco Javier Sánchez-Vázquez, Jose Fernando López-Olmeda, Luisa Maria Vera, Herve Migaud, Marcos Antonio López-Patiño, Jesús M. Míguez

**Affiliations:** ^1^Department Physiology, Faculty of Biology, University of Murcia, Murcia, Spain; ^2^Institute of Aquaculture, University of Stirling, Stirling, United Kingdom; ^3^Laboratory Animal Physiology, Department Biology and Health Science, Faculty of Biology and Centro Singular de Investigación Mariña-ECIMAT, University of Vigo, Vigo, Spain

**Keywords:** daily rhythm, light, temperature, HPI axis, wavelength, thermocycles, fish welfare

## Abstract

Fish have evolved a biological clock to cope with environmental cycles, so they display circadian rhythms in most physiological functions including stress response. Photoperiodic information is transduced by the pineal organ into a rhythmic secretion of melatonin, which is released into the blood circulation with high concentrations at night and low during the day. The melatonin rhythmic profile is under the control of circadian clocks in most fish (except salmonids), and it is considered as an important output of the circadian system, thus modulating most daily behavioral and physiological rhythms. Lighting conditions (intensity and spectrum) change in the underwater environment and affect fish embryo and larvae development: constant light/darkness or red lights can lead to increased malformations and mortality, whereas blue light usually results in best hatching rates and growth performance in marine fish. Many factors display daily rhythms along the hypothalamus-pituitary-interrenal (HPI) axis that controls stress response in fish, including corticotropin-releasing hormone (Crh) and its binding protein (Crhbp), proopiomelanocortin A and B (Pomca and Pomcb), and plasma cortisol, glucose, and lactate. Many of these circadian rhythms are under the control of endogenous molecular clocks, which consist of self-sustained transcriptional-translational feedback loops involving the cyclic expression of circadian clock genes (*clock, bmal, per*, and *cry*) which persists under constant light or darkness. Exposing fish to a stressor can result in altered rhythms of most stress indicators, such as cortisol, glucose, and lactate among others, as well as daily rhythms of most behavioral and physiological functions. In addition, *crh* and *pomca* expression profiles can be affected by other factors such as light spectrum, which strongly influence the expression profile of growth-related (*igf1a, igf2a*) genes. Additionally, the daily cycle of water temperature (warmer at day and cooler at night) is another factor that has to be considered. The response to any acute stressor is not only species dependent, but also depends on the time of the day when the stress occurs: nocturnal species show higher responses when stressed during day time, whereas diurnal fish respond stronger at night. Melatonin administration in fish has sedative effects with a reduction in locomotor activity and cortisol levels, as well as reduced liver glycogen and dopaminergic and serotonergic activities within the hypothalamus. In this paper, we are reviewing the role of environmental cycles and biological clocks on the entrainment of daily rhythms in the HPI axis and stress responses in fish.

## Environmental Cycles and Biological Clocks in Fish

The environment is rarely constant and fluctuates most of the time. Although some environmental changes are unpredictable (e.g., metereological phenomena such as rain or wind), other cyclic fluctuations such as tides, day length, moon phases and seasons are highly predictable. These environmental cycles are governed by geophysical cycles originating from the rotation of the Earth and the Moon around the Sun. Time-keeping systems (i.e., circadian clocks) have evolved since the most primitive forms of life to cope with natural cycles and anticipate periodic events (1). In fish, as in other vertebrates, most behavioral and physiological processes exhibit rhythms, which are driven by molecular clocks made up of transcriptional/translational loops of several clock genes (*per, clock, bmal, cry, ror*, and *reverb*) (2, 3).

Light and temperature cycles are the two main synchronizing signals (so called “zeitgebers” or time-givers) to entrain biological clocks. Light information is transduced into a nocturnal rhythm of melatonin that acts as an internal zeitgeber setting up the phase of individual pacemakers. Daylength, the basis for photoperiodism and seasonality, is coded by the duration (longer/shorter) of the nocturnal melatonin rhythm (4). In addition, light characteristics should be considered, since the underwater photo-environment is peculiar as light is absorbed differently by the water column, so that only blue light (λ ~450 nm) reaches deep marine waters (up to 200 m in clear oceanic waters -euphotic zone), while red light (λ > 600 nm) is quickly absorbed within the first 20 m. Thus, melatonin synthesis is suppressed by light differently depending on the wavelength: shorter (blue) being more effective than longer (red) wavelengths (5). Artificial lights differ greatly from the natural solar light, because classic light bulbs (incandescent filaments) produce a reddish inefficient light underwater, while fluorescent tubes produce sharp peaks at specific wavelengths far from natural daylight. Modern light-emitting diode (LED) technology, however, provides better cost-effective lighting systems which can be used for different purposes in aquatic research (6). Using such technology, light spectrum has been found to affect the ontogeny of the molecular clock, as *clock, per*, and *bmal* gene expression was affected by lighting conditions during early larval development. Furthermore, larvae reared under constant darkness became arrhythmic, while under light/dark cycles of different wavelengths their daily activity rhythms appeared earlier under blue than under white or red lights (7).

The daily day/night alternation not only imposes a light cycle but also a temperature cycle, as the water warms up during the day following sunrise, and cools down at night after sunset. Such a daily thermo-cycle (TC, 12 h cold:12 h warm) synchronizes the circadian clock, which periodicity (tau) is temperature-compensated and remains constant in a wide range of temperatures, with a Q10 value for tau around 1 (8). Actually, clock transcriptional regulatory elements are entrained by TC in embryos and primary cell lines of zebrafish (*Danio rerio*) (9), although light controlled elements (p*er2* and c*ry1a*) do not show rhythmic expression under TC (10). Regarding melatonin, as early reported by Underwood and Calaban (11) in lizards, its rhythmic secretion can be synchronized in constant dark (DD) and constant light (LL) by daily temperature cycles as low as 2°C in amplitude (melatonin peaking during the cold phase). In pike *in vitro* pineal culture, rhythmic melatonin production persisted in TC (10°C:20°C) and DD, which peaked during the hight temperature (12). Nevertheless, TC cycles synchronized with good strength a melatonin rhythm under DD, providing the high temperature coincided with the subjective dark. Synchronization persited, but the rhythm was of lower amplitude when the high temperature was given during the subjective day. In all cases, the TC rhythm didn't entrain the melatonin rhythm as a release into constant temperature resulted in a rapid damping of the melatonin rhythm. As to locomotor activity rhythms, however, under TC and ahemeral light-dark (LD) cycles (conflicting zeitgebers), zebrafish displayed relative coordination, while in constant dim light they synchronized to TC, and they also free-run in constant temperature. These findings indicate that TC alone can entrain zebrafish rhythms, suggesting the participation of both light- and temperature-entrainable oscillators which are weakly coupled (13, 14).

## Phototransduction and Melatonin Rhythms in Fish

Melatonin is a key hormone acting in the circadian system of vertebrates, and it is mainly produced by the pineal gland. In fish, the pineal is a complex structure located in an evagination of the roof of the diencephalon, which exhibits photoreceptive characteristics (15, 16). The pineal epithelium contains photoreceptor cells that resemble the retinal cones of the retina, both on a structural and functional point of view (17–19). These cells elaborate an electrical message at night when they are depolarized, which results in the release of an excitatory neurotransmitter. Meanwhile, light induces hyperpolarization of the photoreceptor cells and inhibits the discharge of the pineal neuronal units (20–22). In addition, as early reported by Falcon et al. (23), photoreceptor cells contains the amino acid (tryptophan) and all the indole compounds (serotonin, N-acetylserotonin, melatonin) and enzymes (see later) to produce melatonin (24–29). The pineal hormone displays daily and seasonal patterns of secretion with elevated levels at night and basal levels during the day, regardless of the fish species studied. Therefore, robust and predictable rhythms of melatonin secreted from the pineal to the blood and likely to the CSF, with which the pineal epithelium communicates in its apical part (30) are expected. The rhythmic melatonin output, which reflects the prevailing photoperiod, is an efficient signal to entrain a wide number of processes that occur at daily and seasonal levels (4).

The synthesis of melatonin also occurs in the retina, which in teleost has been usually, but not exclusively, associated with photoreceptor cells (31–33). Although rhythmic on a daily basis, the pattern of retinal melatonin is substantially different from that in the pineal organ, with melatonin content peaking during the night, or at different times during the day or modifying the phase of the rhythm throughout seasons depending on the species (34–37). Moreover, retinal melatonin is thought to act as a local neuromodulator within the eye (32, 38, 39) and it could be metabolized *in situ* (40), which prevents retinal melatonin to be released to the blood. More doubt arises from a synthesis of the hormone in other body tissues of fish, the intestine being reported to hold relevant amounts of melatonin (41–43). In addition, the presence of mRNA transcripts of melatonin synthesis enzymes has been reported in the digestive tract of several teleost species such as goldfish (44), carp (45), and rainbow trout (43), with daily rhythms that adjust to the prevalent photoperiod. Although a more formal demonstration of melatonin synthesis in fish intestine is needed, it seems like its contribution to plasma melatonin rhythms should be very poor in comparison with the pineal melatonin source, as low night levels or lack of plasma melatonin rhythms are found in pinealectomized fish (43, 46).

Studies in several teleost provide well-founded data about the distribution of melatonin binding sites in wide range of body tissues (47–50). Therefore, this hormone can be involved in multiple physiological processes, most of them displaying daily and/or seasonal rhythms, such as those of locomotor activity, skin pigmentation, food intake, osmoregulation, growth and reproduction [for reviews (3, 4, 51, 52)]. Thus, the melatoninergic output is part of the time-keeping system and enable the fish to synchronize with the closest environment (51). The characteristics of its daily rhythm are well conserved independently on the organization of the system that controls such rhythm. The LD cycle is the prevalent cue that directly or indirectly through the circadian clock system, controls pineal melatonin synthesis and adjust its daily profile in blood (29, 51, 53, 54). The nocturnal rise in melatonin observed in all vertebrates is the consequence of two enzymatic steps that transform serotonin into melatonin: arylalkylamine N-acetyltransferase (AANAT) catalyses serotonin synthesis, whereas hydroxyindol-*O*-methyl transferase (HIOMT) transforms N-acetylserotonin in melatonin (55). In vertebrates, AANAT enzyme is the rate-limiting step for clock-dependent light influence on melatonin synthesis, since this enzymatic activity displays daily oscillations with light inhibiting it during daytime (56). Interestingly, teleost fish, unlike other vertebrates, possess two AANAT subfamilies, namely AANAT1 and AANAT2, which is likely to derive from the whole genome duplication that occurred close the origin of fish (57–59). Whereas, AANAT1, which is homologous with the AANAT found in tetrapods, is expressed preferentially in the retina and discrete brain areas of fish, AANAT2 is more specifically expressed in the pineal gland and has no equivalent in other vertebrates (22, 60).

In contrast to that of mammals, fish pineal photoreceptors cells contain the whole machinery of a light entrained circadian system: photoreceptor unit, clock machinery and melatonin production system (25, 29, 61, 62). Indeed, melatonin synthesis in most teleost species continues to be rhythmic in pineal explants and this rhythm adjusts to a 24-h cycle when they are exposed to a fluctuating light environment (25, 31, 63–65). The connection between pineal clock system and rhythmic melatonin synthesis occurs through a CLOCK-BMAL dimer binding to an *E-box* in the *aanat2* promoter (66–68). Thus, accumulation of *aanat2* mRNA as a result of increased gene transcription during the second half of the day allows AANAT2 protein to be high soon after night onset. Light at the following day resets the clock, which makes AANAT2 enzyme activity and melatonin synthesis to drop (69). The salmonidae lineage, which includes the rainbow trout (*Oncorhynchus mykiss*) and Atlantic salmon (*Salmo salar*), breaks this rule since it lacks an intra-pineal oscillatory mechanism (70). Because of that, rhythmic melatonin synthesis occurs only under an LD cycle both *in vivo* and *in vitro* (71–74). Additionally, melatonin synthesis from fish pineal varies between seasons, which is interpreted by the clock machinery, then modulating annual rhythms (36, 75, 76). Light properties such as intensity and spectrum impact on the amplitude of the melatonin peak, therefore melatonin secretion varies in fish as a result of water depth, time of the day (dawn and dusk), weather conditions, moon phase or latitude (4). Moreover, water temperature is another external factor that acts on the pineal organ to influence melatonin rhythm, through the regulation of AANAT2 activity. A good correlation of AANAT2 activity at night exists for some teleost such as rainbow trout, pike (*Esox lucius*), sea bream (*Sparus aurata*), and zebrafish, with optimal physiological temperatures (12, 29, 60, 72). This strongly supports that both light and temperature act together to provide accurate tuning to daily and annual cycles of melatonin in fish (4, 77).

## Rhythms in the HPI Stress Axis

**Figure 1:**
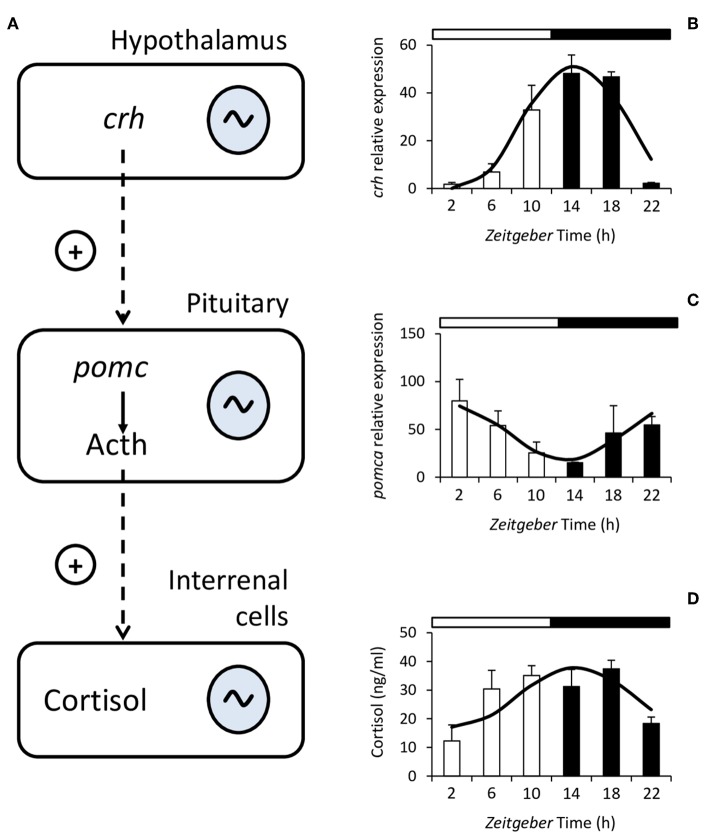
Schematic diagram of the hypothalamus-pituitary-interrenal (HPI) axis **(A)**. Corticotropin-releasing hormone (Crh) is synthesized in the hypothalamus and stimulates, at the pituitary, the synthesis and release of adrenocorticotropic hormone (Acth), which is formed from Proopiomelanocortin (Pomc). Acth stimulates the production and release of cortisol in the interrenal cells. In fish, the HPI axis presents daily rhythms at all of its levels. To the right of the figure, representative examples of the rhythms of *crh* expression **(B)**, *pomca* expression **(C)**, and plasma cortisol **(D)** from Senegalese sole are shown. Mean ± S.E.M. are represented by the bars and errors, the continuous curve represents the cosine function calculated from a significant Cosinor analysis (*p* < 0.05). White and black bars above the graphs represent the light and dark period, respectively. Modified with the permission of authors from López-Olmeda et al. (83).

A wide variety of physiological variables display rhythmicity in fish, among them many factors of the endocrine system such as those produced at all levels of the hypothalamus-pituitary-interrenal (HPI) axis (78, 79), which is the main neuroendocrine circuit involved in the primary response to stress in fish, together with the catecholamine-producing chromaffin cells from the hypothalamic sympathetic nervous system (80, 81). The hypothalamus synthesizes corticotropin-releasing hormone (Crh) which in turn stimulates the synthesis and release of adrenocorticotropic hormone (Acth) from the pituitary (82). Acth is generated from the cleavage of the Proopiomelanocortin (Pomc) and stimulates the production and release of glucocorticoids in the cells of the fish interrenal tissue (82) (Figure 1). The main glucocorticoid produced by fish is cortisol which, besides its main role in the stress response and stress-related homeostasis, influences many other processes such as behavior, growth, reproduction, and osmoregulation (80, 82, 84, 85).

Studies on the rhythmicity of factors from the HPI axis have mainly focused on cortisol, whose daily rhythms have been described in a wide variety of species (78, 86). In addition, daily rhythms have also been reported in other factors from the HPI axis such as the hypothalamic *crh* and pituitary *pomc* gene expression (83, 87, 88). Regarding cortisol, the characteristics of the rhythm such as mesor (similar to the median), amplitude (difference between mesor and highest or lowest point), and acrophase (the time of day when the highest values can be found) are species-dependent. Cortisol rhythms persist under environmental constant conditions, i.e., constant light (LL) or darkness (DD), in some species such as gilthead sea bream, Senegalese sole and rainbow trout (89–91). This persistence in the absence of external cues (free-running) indicates that the rhythm is controlled by circadian oscillators located within the organism (79).

Moreover, besides the daily rhythms that seem to be mainly controlled by variations in the LD cycle, cortisol is also influenced by seasonal variations in photoperiod and water temperature and by feeding time. Annual cortisol variations have been described in several fish species and they seem to correlate mainly with the seasonal reproduction, with the highest annual cortisol levels being located around the spawning season (78). On the other hand, a fixed feeding time can act as the entraining signal of cortisol rhythms in the absence of other external signals such as the LD cycle (92, 93), and different fixed feeding times are able to shift the cortisol rhythm (94). Therefore, the season of the year and the feeding strategy are factors that should be considered when studying cortisol rhythms.

## Stress and Melatonin Interplay in a Rhythmic Environment

Light disturbance either in natural environment, i.e., artificial nighttime lighting, or during farming is another critical factor that could induce stress in animals, including fish (95–97). In this context, studies on environmental stress effects on vertebrate circadian systems are still scarce. In mammals, constant light exposure or food intake out of circadian phase potentially alter the diurnal level of secreted glucocorticoids (GC) and stress-induced GC response (98). Additionally, GC and catecholamines can act as synchronizers of circadian clocks (99, 100). The glucocorticoid receptors (GR) are expressed ubiquitously in nearly all tissues and organs, with the exception of SCN, where no GR expression was noted (99). However, several genomic and non-genomic pathways exist, through which GC can influence circadian core clock genes. In this context, stress at the photophase onset causes a phase-advance of mRNA expression of several core clock genes in peripheral organs (101). Meanwhile, when applied at different times during the photophase, it causes delay or even loss of synchrony, indicating that influence of stress on peripheral clocks depends on the time of day.

In fish, environmental stressors are increasingly related to changes in water conditions including elevated temperature (e.g., global warming or proximity to nuclear plants or cities), presence of pollutants, and oxygen deficits. Routine husbandry in aquaculture also involves further factors, such as stocking conditions, handling, feeding and social interactions, among others (102–104), several of which are also influenced by human intervention. In fish, the effect of stress induced by high density stocking on the daily profile of hypothalamic mRNA abundance of circadian clock genes (*clock1a, bmal1, per1*, and *rev-erb*β*-like*) was recently studied. Decreased amplitude and mean expression levels for most of these genes appeared in stressed trout, except for *rev-erb*β*-like* whose expression increased (105). Furthermore, treatment of trout with the GR antagonist, mifepristone, previously exposed to a stressor failed to prevent these stress-induced changes, suggesting that cortisol is not directly modulating clock gene expression within the hypothalamus in trout. Additionally, this study provides evidence for the involvement of Sirtuin1 (Sirt1), a member of the histone deacetylases family which links cellular metabolism and circadian clocks in mammals (106) and fish (91). Sirt1 deacetylates *bmal1* and *per2* in the liver (107) and activates hypothalamic SCN pacemaker in mice (108). Moreover, *sirt1* mRNA accumulates rhythmically under normal LD conditions and increases sharply in the hypothalamus of stressed trout (105). Therefore, Sirt1 is a good candidate to mediate the effects of stress on the circadian clock genes, not only in peripheral metabolic tissues (liver), but also centrally at the hypothalamic level, where a neuronal network integrates the effects of stress to modulate nutrient sensing information and regulate feeding behavior (109, 110). It is also involved in the regulation of the rhythmic profile of clock genes at the brain level (105), suggesting a role of Sirt1 in the crosstalk between stress response and central circadian system in fish.

The pineal melatoninergic system in vertebrates has been also reported to be influenced by stress and GC treatment in early studies in the 70s [e.g., (111)], and later both *in vitro* (112, 113) and *in vivo* (114–117). In rodents, forced physical activity every 2 h for the 24 h around the clock, results in lower melatonin levels at night, thus flatting normal daily melatonin rhythm (118). Additionally, chronic stress alters the expression of sympathetic markers in rodent pineal gland and increases plasma melatonin concentrations (119). Increased melatonin levels during daytime after immobilization alone or together with dexamethasone treatment were reported in the avian ring dove (*Streptopelia capicola*) (114). A prolonged, but not acute, treatment with dexamethasone also suppressed melatonin production in chick pineal gland and retina, with Aanat activity being significantly lower than that of controls (115). Regarding fish, it seems that pineal melatonin is very sensitive to different environmental stressors, although differences were observed depending on the species and stress type. Rainbow trout initially adapted to freshwater conditions (6 ppt) that were later transferred to isosmotic (12 ppt) and hyperosmotic conditions (18 ppt) showed an increased melatonin content at night in pineal gland and plasma, as compared to the initial status, both in a short-term (6 h) and long-term (5 days) exposure (120). A stimulatory effect of salinity on pineal *aanat2* mRNA abundance and enzyme activity was identified at day- and night-time, with melatonin synthesis enzymes under the regulation of cortisol. This suggests that increased blood osmolality and plasma cortisol levels induced by the hypersaline environment promotes melatonin synthesis in the pineal organ of rainbow trout by increasing Aanat activity independently of the regulatory action exerted by light. In cocho salmon, however, plasma melatonin remain constant during parr to smolt transformation, but increased upon seawater entry (121). Other stressors, like chasing and high-stocking density inhibit melatonin synthesis at night, thus disrupting melatonin rhythms and the capacity of fish to translate environmental information (122). A drop in pineal serotonin content, *aanat2* gene expression, and Aanat enzyme activity was also reported at night. This fits with a diminished N-acetylation pathway as a consequence of lower substrate availability and enzyme activity. In this context, cortisol is likely to have a key role in mediating stress-effects on melatonin synthesis in the pineal organ of trout. In fact, intraperitoneal (IP) cortisol implants reduced melatonin synthesis at night in a similar way than exposure to stressors, and incubation of cultured pineal organs with cortisol reduced melatonin synthesis during the dark phase of the 24-h cycle, with this effect prevented when a GR antagonist was added (113, 122).

Several published studies also support a modulatory role of GC in teleost pineal organ. High cortisol concentrations (100 ng/ml) mimicking stressed conditions were shown to reduce melatonin secretion from cultured pineal organs of tilapia (*Oreochromis mossambicus*) (123), although a similar effect was not observed at night, when cortisol was physiologically elevated in stressed fish. In contrast, socially subordinated rainbow trout displayed concomitant increases in cortisol and melatonin levels in blood, suggesting that social status of the animals may modify the circadian cycles of these hormones. In the North African catfish (*Clarias garieinus)*, treatment with corticosteroid hormones in a μM to mM range inhibited pineal AANAT activity in a dose-dependent way during different phases of the breeding cycle (124). Meanwhile, rainbow trout pineal organs incubated with the GC analog, dexamethasone, at nM concentrations also exhibited inhibition of AANAT2 activity, without affecting HIOMT activity (113). Since a daily variation of *gr* mRNA has been reported in the pineal organ (123) it is plausible that GC effects on melatonin synthesis are modulated by oscillation of GR signaling, which involves the activation of glucocorticoid-responsive elements at the Aanat promoter (113). Alternatively, GC actions are also likely mediated by cell surface receptors that modify Ca2+ and cAMP levels (82), therefore being potentially able to modulate rhythmic melatonin synthesis by the photosensitive pineal cells (4).

In fish, the stress response involves a series of physiological components organized in two neuroendocrine axes, the brain-sympathetic-chromaffin (HSC), and the HPI tissues, whose activation by stressors lead to increased catecholamines and cortisol blood levels, respectively (125). Several studies showed that melatonin might play a role in alleviating stress effects in teleosts, which in many cases relates to the modulation of neuroendocrine responses within the HPI axis. For instance, Munro (126) showed that intracerebroventricular (i.c.v.) injections of melatonin (10 μg) reduced aggressive behavior in the cichlid *Aequidens pulcher* to a mirror presented 20 min later, whereas Larson et al. (127) reported that socially subordinated fish have higher night melatonin levels and no elevation of cortisol levels compared to non-stressed fish. On the other hand, several studies report that treatments with melatonin at doses mimicking nocturnal increase of the hormonal levels were able to reduce stress effects in fish. Thus, melatonin given orally (40–200 mg/g food) or dissolved in water (10 μM) attenuated several effects of chronic stress in rainbow trout (128), and Senegalese sole (*Solea senegalensis*) (129), such as elevated plasma cortisol, inhibited food intake, altered activity of some digestive enzymes, and increased plasma lactate levels and liver glycogenolitic potential (128). Accordingly, Gesto et al. (130) showed that adding melatonin at doses as low as 10 nM into the fish tank was effective in reducing the intensity of stress response induced at short-term by chasing. Thus, a simple treatment with melatonin attenuated the response to cortisol, including the increase of hypothalamic *crh* mRNA content and that of enzymes involved in the steroidogenesis pathways at the head kidney, which normally allow cortisol secretion to increase soon after fish is stressed. Also, intraperitoneal (i.p.) administered melatonin at doses as low as 10 μg/g body weight for 7 days resulted in reduced plasma cortisol levels and locomotor activity of goldfish (*Carassius auratus*) (131), thus suggesting that peripheral melatonin inhibits the stress response and displays additional sedative effects in teleost.

The mechanisms through which melatonin mitigates stress is currently unknown, although both central and peripheral actions of melatonin are suspected to be involved. In fish, the brain serotonergic system is believed to play a role in the activation of the neuroendocrine responses to both acute and chronic stress, including social stress (132–134). An increased serotonergic function starts immediately after exposure to the stressor, particularly affecting the hypothalamus and telencephalon, two regions that receive serotonergic neuronal endings (132, 133). At the level of the hypothalamus-preoptic area, serotonin stimulates the HPI axis by increasing Crh release, which boosts the downstream GC stress response (125, 134). Studies have revealed that melatonin can interact with serotonin to modulate its function (109, 130, 135). Moreover, melatonin ability to reduce stress in teleosts has been usually associated with simultaneous changes in brain serotonergic activity (109, 130, 133). Indeed, melatonin treatment decreased c*rh* mRNA in sole which was upregulated by environmental stressors (130), pointing to a melatonin interplay with serotonin- and Crh-containing neurons in the hypothalamic-preoptic area. Specific studies are lacking to demonstrate the underlying mechanisms of the actions of melatonin on brain serotonin at the cellular level, as well as those that activate the endocrine response to stress. For instance, 5-HT1A-like receptors were involved in mediating increases in *crh* mRNA and Acth hormone secretion in the Gulf toadfish to crowding stress (136) and to modulate HPI axis response in Arctic charr (*Salvelinus alpinus*) (137). This suggests these receptors are potential candidates for serotonin-mediated effects of melatonin to reduce stress response in teleosts, and this hypothesis should be further tested.

Additionally, the possibility that melatonin acts directly on adrenal tissue to modulate GC secretion exists, as reported in mammals (138), and also suggested in fish where i.p., but not i.c.v., melatonin treatment was able to reduce cortisol secretion (131). The presence of melatonin binding sites and mRNA expression of melatonin receptors has been demonstrated in several teleost species (47, 48). Finally, besides applying pharmacological and molecular tools to gain knowledge on the melatonin-cortisol interaction, it is intriguing to know whether the endogenous high levels of melatonin at night are involved in modulating cortisol secretion, either through the HPI axis and interrenal cells or by tuning the daily rhythmic cortisol profile, through the circadian system.

## Light and Temperature Stressors During Early Development and Adulthood

**Figure 2:**
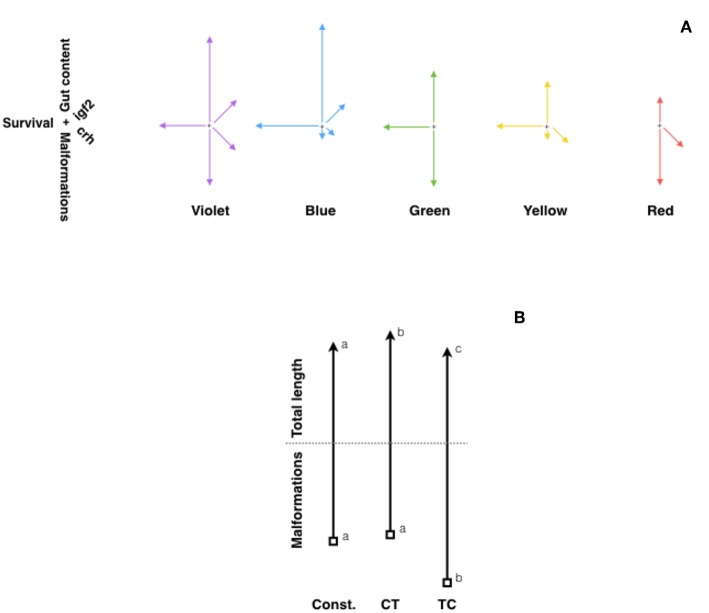
Fitness diagrams of **(A)** zebrafish exposed to different light spectrum (violet, blue, green, yellow, and red), and **(B)** Senegalese sole larvae at 30 DPH raised under constant temperature (21.5°C), or two daily thermocycles: TC (22°C-day:19°C-night) or CT (19°C-day:22°C-night). In **(A)**, lines represent relative values for malformations (vertical, downwards arrow), survival rate (horizontal, left arrow), gut content (vertical, upwards arrow), and expression of *igf2* (rigth-up) and *crh* (right-down) genes. Modified from Villamizar et al. (143). In **(B)**, vertical upwards arrows represent relative values for total length, while downwards arrows represent malformation rates. Different letters indicate significant differences. Modified with the permission of authors from Blanco-Vives et al. (141).

The environment during early life stages permanently alters behavior and physiology by “programming” the expression of selected genes. Actually, environmental stress in early life can impair normal development, predisposing to disease in adulthood (139). Light characteristics (intensity and spectrum) change underwater and affect fish embryo and larvae development (140). In fact, constant light, constant darkness or LD cycles of red lights lead to increased malformations and mortality, whereas LD cycles of blue light produced best hatching rates and growth performance in European sea bass and Senegalese sole (141, 142). In zebrafish, LD cycles of different light wavelengths (violet, blue, green, yellow, red, and white) led also to differences in development, growth, malformations and ultimately survival, upregulating the expression of key genes of the somatotropic (*igf1a* and *igf2a*) and stress axis in fish (crh and pomca) (143). On one hand, growth was enhanced in larvae exposed to LD cycles of violet and blue lights, which showed also significantly higher expression of *igf1* and *igf2*. On the other hand, the LD cycles of violet light produced the highest malformation rates and increased expression of crh, while the best survival rate and feed intake was achieved in fish exposed to LD cycles of blue light (Figure 2A).

Light spectral responses may differ depending on the fish species. In tench, locomotor activity and cortisol levels were influenced by light spectrum, since juvenile tench kept under white and blue lights were less active at night, and cortisol levels were higher in fish kept under white light than in those under constant darkness (144). Fish under red light behaved in a similar fashion as those in darkness. In fact, in some fish species red light may stimulate feeding activity, although such an increase in feeding does not necessarily elicit higher growth. That is the case of Nile tilapia, which showed higher feed intake under red light than under white, blue, green and yellow lights, but failed to show differences in growth rates of feed conversion efficiencies (145). This lack of growth differences despite the increase in food intake maybe related to changes in metabolism, which made food energy being channeled to stress or swimming. In this species, however, blue light prevented confinement stress responses and produced lowest cortisol levels compared to fish under green or white lights (146, 147). In Atlantic cod and turbot (*Scophthalmus maximus*), larvae reared under shorter wavelengths (blue and green lights) showed significantly enhanced growth in comparison to larvae reared under longer wavelengths (red light) (148). Reproduction was also affected by light color, nest construction in Nile tilapia being enhanced under blue light as well (149).

Background color and light contrast are further relevant issues to be considered. In Jundiá (*Rhamdia quelen*), a south american aquacultured fish, the combination of tank color and shelter availability reduced stress responses as cortisol levels decreased in fish kept in talks with blue walls and shelter (150). In the Caspian kutum (*Rutilus frisii*), the color of the tanks (black, blue, red, yellow or white) appeared also to influence food intake and lipid content without changing growth or feed conversion rates (151). Eurasian perch (*Perca fluviatilis*) larvae also showed better growth and prey intake when raised in black tanks compared to gray tanks (152). The combination of different light and wall tank colors affected also the welfare of beluga (*Huso huso*), since red light had a negative impact in growth, while blue light reduced plasma cortisol and glucose (153, 154). In summary, there seems to be a general consensus in different species pointing at shorter wavelengths (blue and green -the ones matching the natural marine underwater photoenvironment) having a positive effect on fish welfare, regardless of their life stage.

The role of temperature regulating fish metabolism, reproduction, development and other adaptive responses has been widely reported (155). Temperature tolerance in fish has been linked with global warming issues (156) and nutritional factors such as dietary lipids (157). As to the effects of daily thermo-cycles (TC) on fish welfare, however, little is known. An early paper by Spieler et al. (158) reported in goldfish that increasing water temperature from 14 to 23°C for 4 h at different times (7, 11, 15, 19, 23, or 3 h) every day resulted in different body weight and gonadalsomatic index. In Senegalese sole, larvae exposed to TC (22°C-day:19°C-night) grew better, showing fastest development and lowest malformation rates, than those raised under constant temperature (20.5°C) or a reversed daily thermocyle (CT, 19°C-day:22°C-night) (141) (Figure 2B). Moreover, in juvenile sole, daily thermocycles proved to affect sex steroid concentrations (higher estradiol in TC fish), sex determination (which occurred earlier in fish under TC) and sex differentiation: fish exposed to TC showing a higher female proportion (71%) than those under CT (18%) or constant temperature (38%) (141). Similar results were obtained in zebrafish larvae kept under two constant (24°C and 28°C) and two daily thermocycles: 28°C-day:24°C-night (TC) and 24°C-day:28°C-night (CT), embryo development and larval growth being fastest under 28°C and TC, which also showed the highest survival and lowest malformation rates (159). Moreover, in that report sex ratio was also strongly affected by the temperature regime, so that CT and TC produced more females (around 80%), and highest expression of ovarian aromatase (*cyp19a*), which converts androgens into estrogens and thus led to female differentiation.

Acclimation to a cyclic thermal environment can increase thermal tolerance, particularly during early development since the thermal history of larvae induces irreversible changes. As reported by Schaefer and Ryan (160), fish zebrafish larvae reared under daily thermocycles (28 ± 6°C) showed greater tolerance than those reared under constant (28°C) or stochastic (random variations, mean 28°C) temperature regimes. Ongoing research further support these observations as zebrafish larvae challenged to cold/heat shocks (16°C/36°C, respectively) showed reduced mortality rates and enhanced expression of heat shock protein (*hsp70*) when reared under a daily thermocycle as compared to a constant rearing temperature (de Alba et al. unpublished).

## Time-Dependent Stress Responses and Detoxification Rhythms

The endocrine system of fish responds differently depending on the time of the day. For instance, daily differences have been reported in the response to exogenous treatments that affect endocrine pathways controlled by the hypothalamus-pituitary (HP) system such as the administration of exogenous Gh or Gnrh agonists (Gnrha) (161–163).

**Figure 3:**
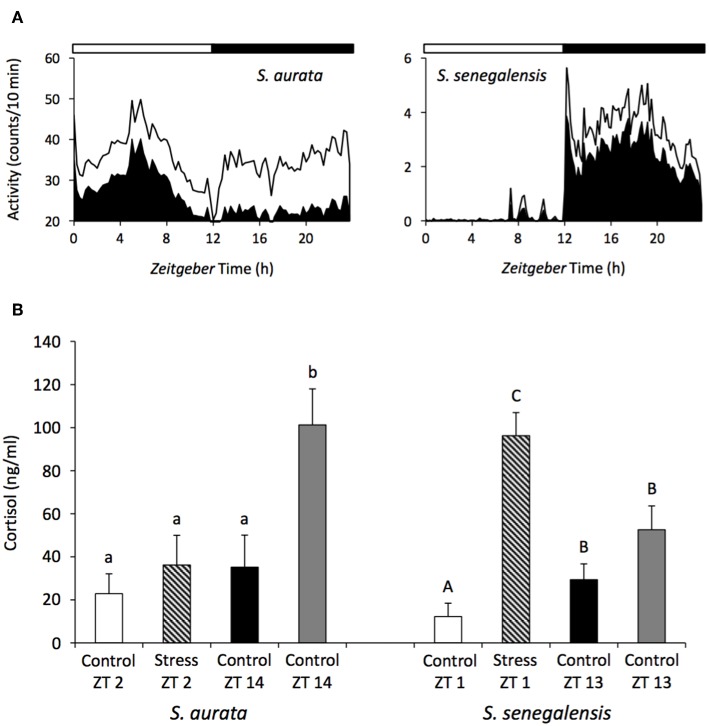
Daily rhythms of locomotor activity **(A)** and differences in the cortisol response depending on the time of the day **(B)** in the gilthead sea bream and Senegalese sole. The black area in the waveforms represents the mean values of activity and the continuous line the S.D. White and black bars above the waveforms represent the light and dark period, respectively. A stress challenge was applied to both species, consisting of air exposure during 30 s, at different time points of the LD cycle: ZT2 and 14 h for sea bream, and ZT1 and 13 h for sole. Fish were sampled 1 h after the stress and cortisol was evaluated. Unstressed control groups were sampled at all-time points. Different letters indicated significant differences between groups (ANOVA, *p* < 0.05) (small case letter for sea bream and upper case letters for sole). Modified with the permission of authors from López-Olmeda et al. (83) and Vera et al. (88).

This different response depending on the time of the day has been reported for the stress response in several fish species such as the green sturgeon (*Acipenser medirostris*), Senegalese sole, gilthead sea bream and African sharptooth catfish (*Clarias gariepinus*) (83, 88, 164–166). Senegalese sole subjected to an acute stress (air exposure) showed a greater cortisol production when the stress was applied at the beginning of the light phase as opposed to beginning of the dark phase (83) (Figure 3). Likewise, a similar stress applied to gilthead seabream at several time points throughout the 24-h cycle elicited greater cortisol responses during darkness compared with the light phase (88) (Figure 3). The daily patterns of locomotor behavior could be partially responsible for the species-dependent differences. Actually, a greater stress response was associated with the resting phase of the species: nocturnal sole presented higher stress during the day, while diurnal gilthead sea bream were more stressed during the night. This hypothesis should be further tested in different fish species, particularly in fish with dual phasing behavior (changing from diurnal to nocturnal) such as sea bass.

The effectiveness of drug absorption, administration, metabolism and elimination are also subjected to rhythmicity, which affects the final concentration of xenobiotics in the animals' blood and their bioavailability (167). In mammals, the existence of toxicity rhythms is widely accepted but in fish species, data remains scarce with only a few studies recently published. In particular, the time-dependent effect of several substances frequently used in aquaculture has been assessed, including anesthetics and veterinary medicines.

**Figure 4:**
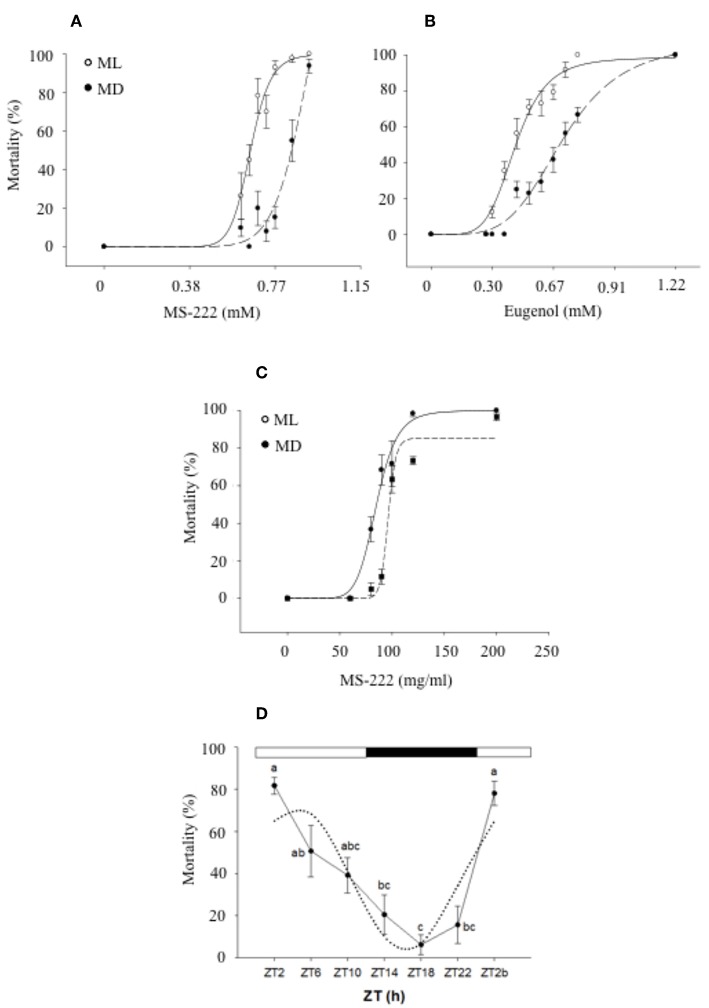
Daily variations of mortality of zebrafish exposed to different MS-222 **(A)** and eugenol **(B)** concentrations after 15 min exposure at mid-light (ML; white circles) or mid-dark (MD; black circles) [with the permission of authors from Sánchez-Vázquez et al. (171)]. Sea bream mortality after 15 min exposure to different MS-222 **(C)** concentrations at ML or MD [with the permission of authors from Vera et al. (172)]. A logistic curve (dotted lines) was fitted to mortality rate (six independent replicates with *n* = 8). **(D)** Daily rhythm of mortality of zebrafish larvae exposed to 5% ethanol for 1 h. Different letters indicate significant differences (ANOVA I, *p* < 0.05), while the dotted black line represents the sinusoidal function fit (Cosinor analysis, *p* < 0.05).

Anesthetics are administered to fish to immobilize them and minimize their stress response during research and routine procedures in fish farms (168). However, anesthetics need to fulfill a number of criteria before being approved for their use in aquatic animals and consequently, toxicology tests have to be performed to determine any toxic effects as well as the optimal concentration required to induce anesthesia, which will be species and temperature specific (169). In this context, it is also important to determine whether the time of administration can have an impact on the effect of these substances. In the case of tricaine methanesulfonate (MS-222), a licensed anesthetic for use in food sources, a daily rhythm of toxicity and effectiveness has been reported in gilthead sea bream (170) and zebrafish (171). In both species, a strong effect of the time of administration was found, with higher toxicity and effectiveness of MS-222 when fish were exposed during the day than at night. In the case of sea bream, the median lethal concentration (LC50) at mid-darkness (MD) was 25.7% higher than at mid-light (ML). In order to determine the induction time of anesthesia at ML and MD, fish were also exposed to sublethal concentrations of MS-222, which revealed that during the day the activity of fish significantly decreased after 7 min of exposure whereas at night no effect was observed until fish had been exposed for 9 min. In addition, the recovery time was longer during the day (10 min) than at night (6 min) (170). These differences in the toxicological response of sea bream were correlated to higher plasma concentrations of MS-222, measured post-exposure, during the day than at night, suggesting a link between the plasma anesthetic levels and the degree of toxicity (172). In zebrafish, similar day-night differences in the effect of anesthetics (MS-222 and eugenol) were found. When fish were exposed to 190 mg/L of MS-222, the mortality rate was 82% at ML whereas at MD this rate descended to 14%. In the case of eugenol, a concentration of 80 mg/L also resulted in a higher mortality rate at ML than at MD (68 and 22%, respectively) which correlated with a shorter induction time of anesthesia during the day (171) (Figure 4). The authors of these studies concluded that toxicity rhythms may be related to the animal's daily pattern of activity. Higher toxicity/effectiveness of anesthetics was observed during the active phase of fish, possibly due to an increase of the ventilatory frequency and as a result, increased uptake of the xenobiotic from the water (170–172).

In Atlantic salmon, the time-dependent effects of hydrogen peroxide have also been investigated. Hydrogen peroxide is a veterinary medicine commonly used to treat ectoparasites such as sea lice (*Lepeophtheirus salmonis*) and amoebic gill disease (AGD) caused by *Neoparamoeba perurans*, but these treatments can have side effects on fish and trigger a stress response following exposure leading to increased mortalities in some cases (173). However, the stress response showed daily rhythmicity in salmon, with cortisol, glucose and lactate levels showing higher levels when the fish were treated during the day than at night (174). In addition, these authors also investigated the effect of hydrogen peroxide on the oxidative stress response in liver, reporting that gene expression of key antioxidant enzymes (*gpx1, cat, hsp70*, and *mn-sod*) was up-regulated when fish were treated during the first half of the day, and in particular around 6 h after the lights onset (175).

In vertebrates, the liver is the main organ involved in detoxification, a process that includes multiple biochemical steps that convert lipophilic toxins into water-soluble metabolites that can then be eliminated from the organism via the urine (176). This system relies on a number of biotransformation enzymes and transporter proteins (177), some of which are regulated by the circadian clock in mammals (178). In zebrafish, recent investigations have revealed that both detoxification genes and key transcription factors regulating their expression are also subjected to circadian control. In particular, the expression of hepatic PAR bZIP proteins (*tefa, tefb, dbpa*, and *dbpb*) and nuclear receptors (*ahr2*) showed daily and circadian rhythmicity, in tune with clock genes expression. These transcription factors and nuclear receptors regulate the expression of many detoxifying enzymes and ABC transporters, some of them also displaying rhythmicity in this species (*cyp1a, gstr1, mgst3a, sult2_st2, abcg2, abcb4, smtb*) (179). Altogether, this study provided evidence about the molecular mechanisms underlying the toxicity rhythms described before in fish species and suggested the existence of clock-control in their toxicological response.

The application of this field of research is evident when designing health strategies in the aquaculture industry. However, it is also important to highlight that zebrafish has become an animal model widely used in biomedical research, to assess the psychoactive and toxic effects of many drugs (180, 181), including the neurobehavioural effects of ethanol (182). Therefore, it is crucial to understand the effect of time of administration when designing these tests. In this context, recent research has revealed a daily rhythm in the effects of ethanol in zebrafish, characterized by higher mortality rates in larvae exposed to 5% ethanol at the beginning of the day (80%) than in the middle of the night (6%). In addition, behavioral effects in adults exposed to 1% ethanol were also more severe during the day, with key genes involved in ethanol detoxification in the liver showing circadian rhythmicity in continuous darkness (DD) (183).

In conclusion, fish chronotoxicity is a novel area of research that is showing promising prospects for the application of chronobiology concepts to optimize the administration of medicines in fish farms, which can lead to improve welfare of animals in commercial settings. Furthermore, increasing our knowledge about toxicity rhythms of drugs used in biomedical research will also have an impact on the application of therapies in humans.

## Photodamage in the Retina

Although light is essential for vision, the trade-off is the production of reactive oxygen species (ROS) that can cause damage within the eye (184). In vertebrates, the negative effect of abnormal light conditions on the retina has been well reported, including studies in fish species. The existence of LD cycles is the most important environmental factor acting as a synchroniser of biological rhythms in vertebrates. For this reason, lighting conditions and photoperiod have been frequently used and manipulated in aquaculture to control the timing of reproduction, overcoming the problems associated with early maturation, such as reduced growth and feed efficiency (185, 186). In particular, continuous light (LL) conditions are commonly used during the production cycle of commercially relevant fish species to control the onset of puberty, increase growth rates, manipulate smoltification in salmonids and improve larvae performances (187–190). However, the use of artificial light sources and regimes can also have a negative impact on fish physiology at different levels, triggering the stress response through activation of the HPI axis, affecting the immune function and inducing retinal damage (191).

The effect of artificial light regimes during early development can be particularly detrimental to fish and have negative effects later during their life cycle. In zebrafish larvae, exposure to abnormal light-rearing conditions (LL or DD) affects their visual behavior and adversely influence the physiological development of the retina, as measured with electroretinogram (ERG) (192). However, artificial lighting systems are used throughout the production cycle in the aquaculture industry. Therefore, lights effects need to be evaluated at different stages of the fish life cycle, especially in those species showing phototactic behavior, as these fish would be exposed to high levels of irradiance when swimming close to the light source (193).

The use of LED technology has increased considerably in the last few years. LEDs have low electrical running costs, a long-life span and can be manufactured to yield specific wavelengths that can be modified according to a species' environmental requirements (194–196). However, the potential adverse effects of these light systems need to be assessed before implementing their use in aquaculture settings. To this end, several studies have focused on these effects in different fish species. In Atlantic salmon, Migaud et al. (191) exposed post-smolt fish to high intensity white and blue LED lights (LL) and investigated their effect on retinal morphology. The study found that high intensity LEDs did not cause retinal damage although the blue lights triggered a stress response in salmon. Similarly, when Atlantic cod were exposed to metal halide (LL, 16.58 ± 8.77 W/m^2^), high green cathode lights (LL, 0.82 ± 0.15 W/m^2^) or low green cathode lights (LL, 0.47 ± 0.18 W/m^2^), no differences in the outer nuclear layer (ONL) thickness or ONL nuclei number were found between groups or in comparison to the control fish under simulated natural photoperiod (SNP, 0.08 ± 0.03 W/m^2^) (197). However, when halogen lights were used, the exposure to continuous high intensity illumination resulted in the induction of retinal damage in Atlantic salmon (*Salmo salar*), Atlantic cod and European sea bass (198). This damage was characterized by morphological alterations that included higher melanin density, forming granules around the photoreceptor cells, photoreceptor necrosis and clear disorganization within the ONL. Interestingly, inter-species differences were found, with cod being the most sensitive species and sea bass the least (cod > salmon > sea bass). Regional variations in the effect of light on the ONL thickness and nuclei were also observed, with the central region of the retina presenting more acute damage. When fish were returned to a LD cycle, retinal regeneration occurred in the three species although the recovery time was also species-specific. Thus, cod showed retinal regeneration after 15 days in LD whereas at least 30 days were needed to observe the same effect in salmon and sea bass (198). In albino zebrafish, exposure to constant intense light also resulted in photoreceptor cell death in the central and dorsal retina, whereas many rods and cods were not affected in the ventral area. In addition, high levels of cell proliferation in both the ONL and inner nuclear layer (INL) were observed, suggesting a potential compensation for the photoreceptors loss, with large numbers of PCNA (Proliferating Cell Nuclear Antigen)-positive cells localized in these layers, indicating a correlation between the magnitude of retinal damage and cell proliferation response (199). In normally pigmented individuals, similar results were found, with high light intensity causing extensive photoreceptor apoptosis and progenitor cell degeneration, mainly in the dorsal and central retinas. In particular, retinal damage triggered Müller glial dedifferentiation and proliferation response of progenitor cells that then migrated to the ONL (200).

Melatonin is also synthesized in the retina of teleost fish, showing marked daily rhythmicity. However, an inverse melatonin profile has been observed in plasma and eye in some fish species, which could be explained by the existence of two different AANAT isoforms and suggests a local function for ocular melatonin (201). One of these roles may be related to the antioxidant properties of this molecule, which can act as a free radical scavenger and also as an anti-apoptotic compound in the retina (202). Actually, recent studies in mammals have concluded that melatonin reduces and even inhibits retinal damage associated to oxidative stress. This anti-apoptotic function could be linked to the inducing effect of melatonin on antioxidant enzymes, as well as its suppressing effect on pro-oxidant compounds (203). In fish, the neuroprotective effect of melatonin against oxidative stress in the retina has not been evaluated yet. However, the antioxidant properties of this indolamine and the fact that its production in the eye of some fish species is higher during the day [reviewed by (204)] suggests that melatonin may play a role in protecting cells against retinal photodamage. Further investigations will be needed to prove this hypothesis.

In summary, there is ample scientific evidence that the use of artificial lights and protocols can induce retinal damage in fish, although important differences between light sources and species have been reported. For this reason, it is crucial to develop and test novel illumination technologies before their implementation in aquaculture systems, to ensure that animal welfare is not compromised. In addition, further studies on melatonin effects in the fish retina will be important to enable us to better understand the cellular mechanisms of retinal photodamage and elucidate whether this hormone play a role as a neuroprotector against light-induced oxidative stress in fish.

## Concluding Remarks and Practical Issues

Fish physiology is mainly rhythmic, governed by biological clocks which synchronizes to the (cyclic) environment in order to improve fitness and ultimately survival. Thus, stress responses in fish are not always straight forward, as they may respond differently on a time-dependent basis. Fish in captivity are challenged by many stressors and the chronobiological approach depicted here should be considered to improve their welfare. For instance, in farming conditions fish should be manipulated at the times when stress is better tolerated, whereas anesthetics and medicines should be used at the optimal times to enhance their efficacy while minimizing toxicity and side effects. Finally, keeping conditions regarding light spectrum and temperature cycles, should be also considered with care, particularly during early embryo and larval development as they may have long lasting irreversible effects. Light contamination at night should be particularly avoided, providing fish with a “melatonin friendly” environment.

## Author Contributions

FS-V, JL-O, and LV provided the figures. All authors contributed equally in the writing and revision of the manuscript.

### Conflict of Interest Statement

The authors declare that the research was conducted in the absence of any commercial or financial relationships that could be construed as a potential conflict of interest.
